# The Sec63p J-Domain Is Required for ERAD of Soluble Proteins in Yeast

**DOI:** 10.1371/journal.pone.0082058

**Published:** 2013-12-04

**Authors:** Christina Servas, Karin Römisch

**Affiliations:** Department of Microbiology, Faculty of Natural Sciences and Technology VIII, Saarland University, Saarbrücken, Germany; Cambridge University, United Kingdom

## Abstract

How misfolded proteins are exported from the ER to the cytosol for degradation (ER-associated Degradation, ERAD) and which proteins are participating in this process is not understood. Several studies using a single, leaky mutant indicated that Sec63p might be involved in ERAD. More recently, Sec63p was also found strongly associated with proteasomes attached to the protein-conducting channel in the ER membrane which presumably form part of the export machinery. These observations prompted us to reinvestigate the role of Sec63p in ERAD by generating new mutants which were selected in a screen monitoring the intracellular accumulation of the ERAD substrate CPY*. We show that a mutation in the DnaJ-domain of Sec63p causes a defect in ERAD, whereas mutations in the Brl, acidic, and transmembrane domains only affect protein import into the ER. Unexpectedly, mutations in the acidic domain which mediates interaction of Sec63p with Sec62p also caused defects in cotranslational import. In contrast to mammalian cells where *SEC63* expression levels affect steady-state levels of multi-spanning transmembrane proteins, the *sec63* J-domain mutant was only defective in ERAD of soluble substrates.

## Introduction

The Sec63 protein is a subunit of the protein translocation channel in the endoplasmic reticulum (ER) membrane which is essential in the biogenesis of secretory and transmembrane proteins in eukaryotic cells [1]. In yeast, Sec63p is a subunit of the heterotetrameric Sec63 complex (Sec63p, Sec62p, Sec71p, Sec72p) which associates with the Sec61 protein translocation channel in the ER membrane to promote posttranslational import of secretory proteins into the ER lumen [1]. The Sec63 complex is also involved in karyogamy in yeast [2]. Sec63p on its own plays a poorly defined role in cotranslational protein import into the ER in yeast [3], [4], [5]. Both Sec62p and Sec63p have homologues in mammalian cells [6], [7]. Mutations in human *SEC63* lead to polycystic liver disease which in its initial stages is characterized by dilated ER cisternae indicative of accumulation of proteins in the ER lumen [8]. A similar phenotype is observed in zebrafish with mutations in *SEC63* [9].

**Figure 1:**
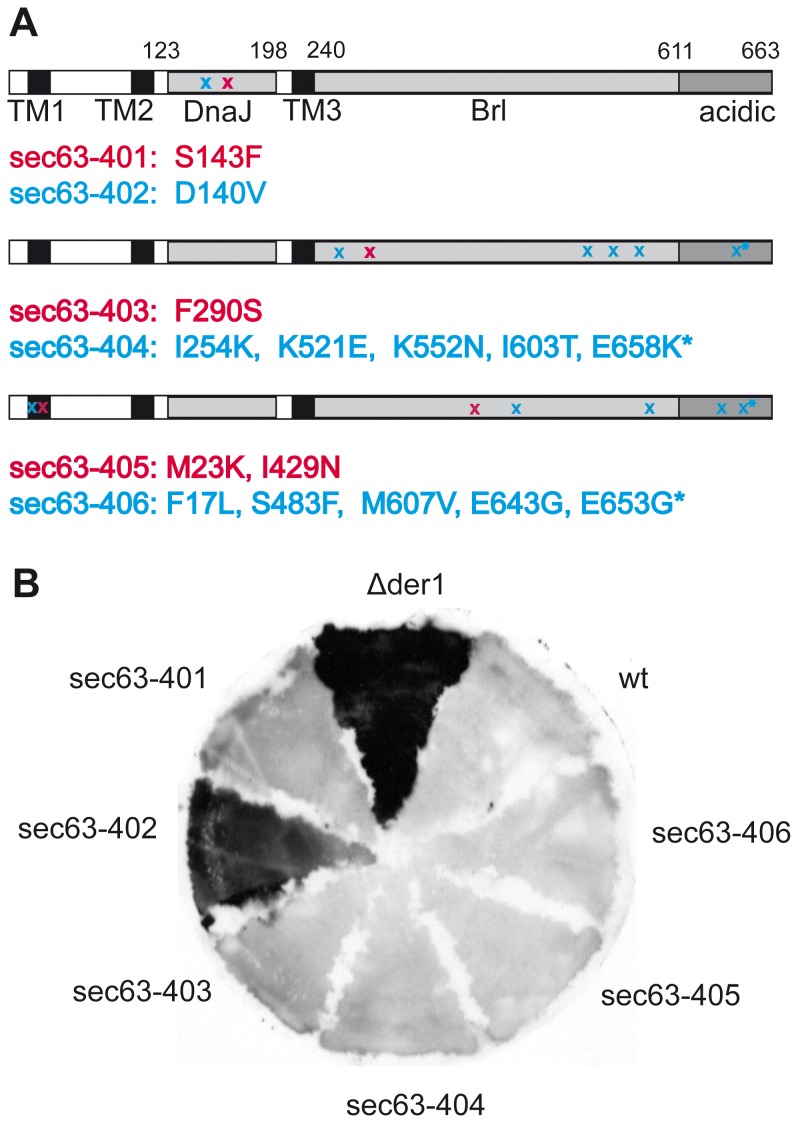
Isolation of new *sec63* mutants using an ERAD-substrate accumulation screen. A: Schematic depiction of 6 new isolated *sec63* mutants. Three transmembrane domains (TM1-3) as well as DnaJ, the Brl and acidic domains are indicated. The respective positions of the point mutations in the mutants are marked with x. Location of mutations in two mutants are shown per drawing in magenta and blue, respectively. Point mutations located in the interaction area with Sec62p are marked with *.B: New *sec63* mutants and control strains *Δder1* (positive control) and wildtype (wt, *SEC63-URA3*-pRS316, negative control) were analysed for CPY* accumulation by colony blotting: yeast were grown on a nitrocellulose membrane on top of a YPD plate, transferred to sporulation media overnight, followed by sporulation media including cycloheximide for 10 h; then cells were lysed and CPY* detected on the membrane with an antibody against CPY.

Sec63p structure has been investigated in the yeast protein. Sec63p is a transmembrane ER protein with 3 transmembrane domains (TMDs), its N-terminus in the ER lumen and its long C-terminus in the cytosol (Fig. 1A) [10]. The C-terminus has a C-terminal acidic domain which is essential for the association with Sec62p [10], [11]. The Sec63p C-terminus is also stably phosphorylated, and the modification enhances its interaction with the N-terminus of Sec62p [12]. Truncation of the acidic domain or mutation of the phosphorylation sites lead to defects in posttranslational protein import into the yeast ER [10], [11], [12]. The Brl domain preceding the acidic domain has been shown to mediate interaction of Sec63p with the Sec61 channel [13]. The ER-lumenal domain between TMD2 and TMD3 is a so-called J-domain which acts as a cochaperone for the ER-lumenal Hsp70 Kar2p (BiP in mammalian cells) and enhances its ATP-hydrolysis [10], [14]. Sec63p J-domain function is essential for posttranslational import into the yeast ER [15], [16], [17].

Proteins that misfold in the ER lumen are transported back to the cytosol for degradation by proteasomes, a process called ER-associated degradation (ERAD) [1], [18]. The identity of the protein translocation channel for ERAD is controversial, but the majority of data indicate that transport of misfolded proteins is mediated by the Sec61 channel [1]. The Sec63 complex is definitely not involved, because Sec62p, Sec71p, and Sec72p are dispensable for ERAD [19], [20]. Whether or not Sec63p on its own is part of the retrotranslocation channel remains controversial: a temperature-sensitive mutation in the Sec63p J-domain, *sec63-1*, led to a 2-fold increase in half-live of two different soluble ERAD substrates, a mutant form of the pheromone precursor prepro-alpha factor (▵gpaF) and a mutant form of the vacuolar protease carboxypeptidase Y (CPY*) [19], [20]. Because the *sec63-1* mutant is leaky, and the cells are compromised for translocation into the ER even at the permissive temperature, it remains unclear whether the effect of the mutation indicated a direct involvement of Sec63p in ERAD, or whether the effect was indirect. In the gene encoding the ER-lumenal Hsp70 Kar2p specific mutations had been identified that cause ERAD defects [21]. In addition, two other ER-lumenal J-proteins, Jem1p and Scj1p, are required for ERAD [22]. Furthermore, we found that Sec63p, but none of the other subunits of the Sec63 complex, is associated with a large complex comprising the proteasome, the Sec61 channel, and the Hrd1p ubiquitin ligase, which likely represents the retrograde protein translocation complex for ERAD [23]. These observations in addition to the dilated ER cisternae in polycystic liver disease patients and *sec63* mutant zebrafish likely indicative of misfolded protein accumulation in the ER prompted us to investigate the role of Sec63p in ERAD in more depth [8], [9].

In this paper we used a screening method that had been applied successfully in the past to isolate genes essential for ERAD [24]. We mutagenized yeast *SEC63* and screened for *sec63* mutants accumulating CPY* using colony blots [24]. We identified several new *sec63* mutants which accumulated CPY*. Upon separation of the mutations in the individual Sec63p domains we found that only mutations in the Sec63p J-domain led to CPY* accumulation. Our J-domain mutant with the strongest ERAD phenotype, *sec63-402*, grew well at the permissive temperature, had no cotranslational, and only a modest posttranslational protein import defect into the ER, but was tunicamycin hypersensitive and displayed a strongly induced Unfolded Protein Response (UPR) indicative of a disturbance of ER protein homeostasis [25]. We conclude that the J-domain of Sec63p plays an active role in ERAD.

## Results

### Isolation of *sec63* Mutants Defective in ERAD

The role of Sec63p in ERAD has been controversial, but only one mutant allele, *sec63-1*, has been investigated for ERAD defects so far [19], [20]. To clarify the contribution of Sec63p to ERAD we screened for *sec63* mutants that accumulated the established ERAD substrate CPY* [24]. We generated random point mutations in *SEC63* by error-prone PCR. Mutant *sec63* genes were cloned into pRS315 (*LEU2*) and transformed into a strain in which the only copy of the essential *SEC63* gene was encoded by a plasmid, pRS316 (*URA3*). The strain also carried a chromosomal copy of the *prc1-1* allele encoding mutant misfolded CPY* [26], [27]. The *SEC63-URA3* plasmid was counterselected on 5-FOA media and the *sec63* mutants were examined by colony blotting for intracellular accumulation of CPY* [24]. As a positive control we used a strain deleted for *DER1*, a gene essential for CPY* degradation [24].

From 4000 colonies we isolated six *sec63* mutants that accumulated CPY* (Fig. 1A, B). Mutants were verified by sequencing the *sec63* alleles on the mutagenized plasmids isolated from these clones. Of these, only *sec63-402* displayed CPY* accumulation comparable to the ▵*der1* strain (Fig.1B). The *sec63-402* mutant carries a point mutation in the ER-lumenal DnaJ domain; this domain mediates interaction of Sec63p with the Hsp70 chaperone Kar2p in the ER lumen (Fig. 1A, top) [16]. A second mutant we isolated, *sec63-401*, has a point mutation in the DnaJ domain as well, but its CPY* accumulation was much more modest (Fig. 1A, top, Fig. 1B). The remaining new *sec63* mutants have one to five point mutations spread throughout the *SEC63* gene (Fig. 1A) which caused only marginal accumulation of CPY* (Fig. 1B). The *sec63-404* and *sec63-406* mutants have five and four mutations, respectively, in the cytosolic C-terminal part that includes the Brl and the acidic domain (Fig. 1A) whereas the two mutations in *sec63-405* are located in the first transmembrane domain and in the Brl domain, and *sec63-403* carries a single point mutation in the Brl domain (Fig. 1A). The last 14 aa of the Sec63p acidic domain mediate the interaction with Sec62p which is important for formation of the Sec63 complex and posttranslational protein import into the ER [28], [29]. The interaction of Sec63p with the Sec61 channel is mediated by the Brl domain [30]. Depending on the location of the individual mutations, our *sec63-403* to *sec63-406* mutants may therefore have defects in the interaction with Sec62p or the Sec61 channel or both. Only the D140V substitution in the DnaJ domain of the *sec63-402* mutant, however, caused a significant intracellular accumulation of CPY* suggesting that this domain is important for ERAD (Fig. 1B).

### Mutations in *SEC63* Differentially Affect Growth and Tunicamycin-Sensitivity

**Figure 2:**
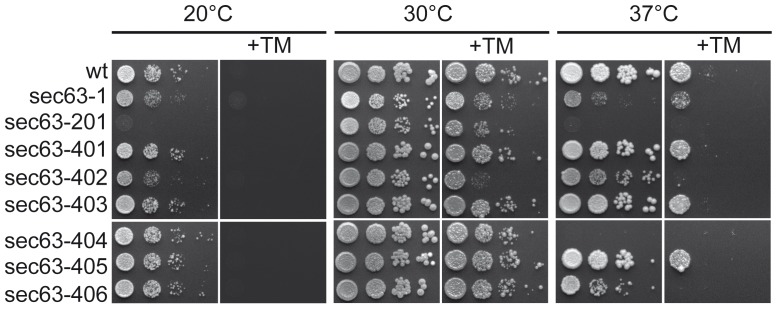
Temperature- & tunicamycin sensitivity of the new *sec63* mutants. 10^1^-10^4^ cells of each *sec63* mutant as well as the corresponding wildtype (wt), and the *sec63-1* and *sec63-201* mutants were grown on YPD plates without or with 0.25 µg/ml tunicamycin at the indicated temperatures. 3 independent experiments were performed.

The ER protein import function of Sec63p is essential, and mutations in ER protein translocation channel subunits frequently lead to temperature- or cold-sensitivity [31], [32]. Yeast mutants defective in ERAD, on the other hand, are usually sensitive to the N-glycosylation inhibitor tunicamycin which increases protein misfolding in the ER [33], [34]. We therefore examined growth of our new *sec63* mutants at various temperatures, and in the presence of tunicamycin. As controls we used the first identified *sec63* mutant, *sec63-1,* which has a point mutation (A179T) in the DnaJ domain and is defective in posttranslational protein import into the ER and ERAD, and *sec63-201* in which the C- terminal 27 amino acids are deleted and which has been shown to be defective in protein translocation into the ER and karyogamy [2], [32]. The strains were grown on YPD at 20°C, 30°C and 37°C. As shown in Fig. 2, both *sec63-1* and *sec63-201* displayed growth defects even at 30°C which were exacerbated at both lower and higher temperatures (Fig. 2). In contrast, the *sec63* mutants isolated in this study grew similar to wildtype at 30°C (Fig. 2, center). At 20°C only the growth of *sec63-402* was compromised compared to wildtype (Fig. 2, left). At 37°C *sec63-404* was unable to grow, and growth of *sec63-402* and *sec63-406* was significantly reduced compared to wildtype, whereas growth of *sec63-401* and *sec63-405* was not affected at this temperature (Fig. 2, right). All *sec63* mutants whose growth was compromised at 37°C were unable to grow in the presence of tunicamycin at this temperature (Fig. 2, right, +TM). At 30°C, however, *sec63-402* was the only one of our mutants that displayed tunicamycin-sensitivity (Fig. 2, center, +TM). Both *sec63-1* and *sec63-201* cells were also sensitive to tunicamycin at 30°C, but not to the same extent as *sec63-402* (Fig. 2, center, +TM). We have shown here that *sec63-402,* the mutant with the strongest CPY* accumulation phenotype, also displays the strongest tunicamycin-sensitivity of all known *sec63* mutants, suggesting a significant defect in ER homeostasis.

To further investigate the influence of the localization of the mutations on temperature sensitivity, we subcloned individual mutated domains into wildtype *SEC63* such that the mutations were restricted to the transmembrane domains including the DnaJ domain, the acidic domain, or the Brl domain (Fig. 1A). These domain-specific *sec63* mutants were grown at different temperatures in the absence or presence of tunicamycin as above (Fig. S1). In *sec63-404* both the mutations in the Brl domain and in the acidic domain contribute to the sensitivity to high temperature (Fig. S1). In *sec63-406*, however, only the mutations in the acidic domain were responsible for the temperature sensitivity (Fig. S1). Growth of *sec63-405* yeast was not affected at any temperature (Fig. 2), and separation of the mutations in transmembrane domain 1 and the Brl domain (Fig. 1A), did not alter their growth phenotype (not shown). Our data indicate that mutations in the cytosolic C-terminal part of Sec63p, especially in the acidic domain, are responsible for the growth defects of our *sec63* mutants at higher temperatures.

### Induction of the Unfolded Protein Response (UPR) in *sec63-402* Cells

**Figure 3:**
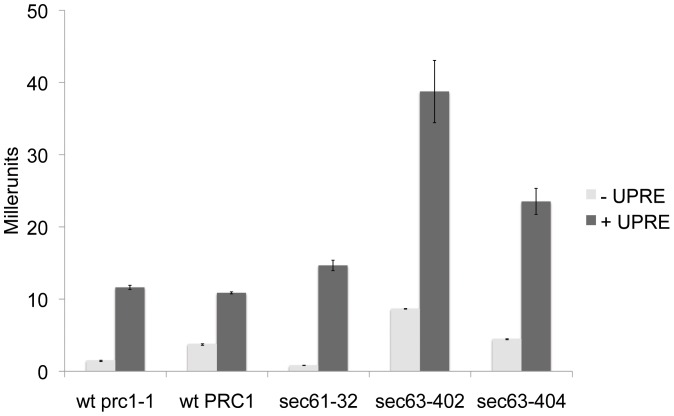
The UPR is strongly activated in *sec63-402*. Mutants *sec63-402*, *sec63-404*, the negative control strains *SEC63-URA3*-pRS316 *prc1-1* and *SEC63-URA3*-pRS316 *PRC1* as well as the positive control *sec61-32* were transformed with plasmids containing *UPRE-LacZ* (pJC31; light grey) or the *LacZ* control (pJC30; black). Cell lysates were incubated with the colorigenic ONPG substrate at 28°C for 20 min. The reaction was stopped and absorption was detected at 420 nm. Standard deviation is indicated in the figure.

**Figure 4:**
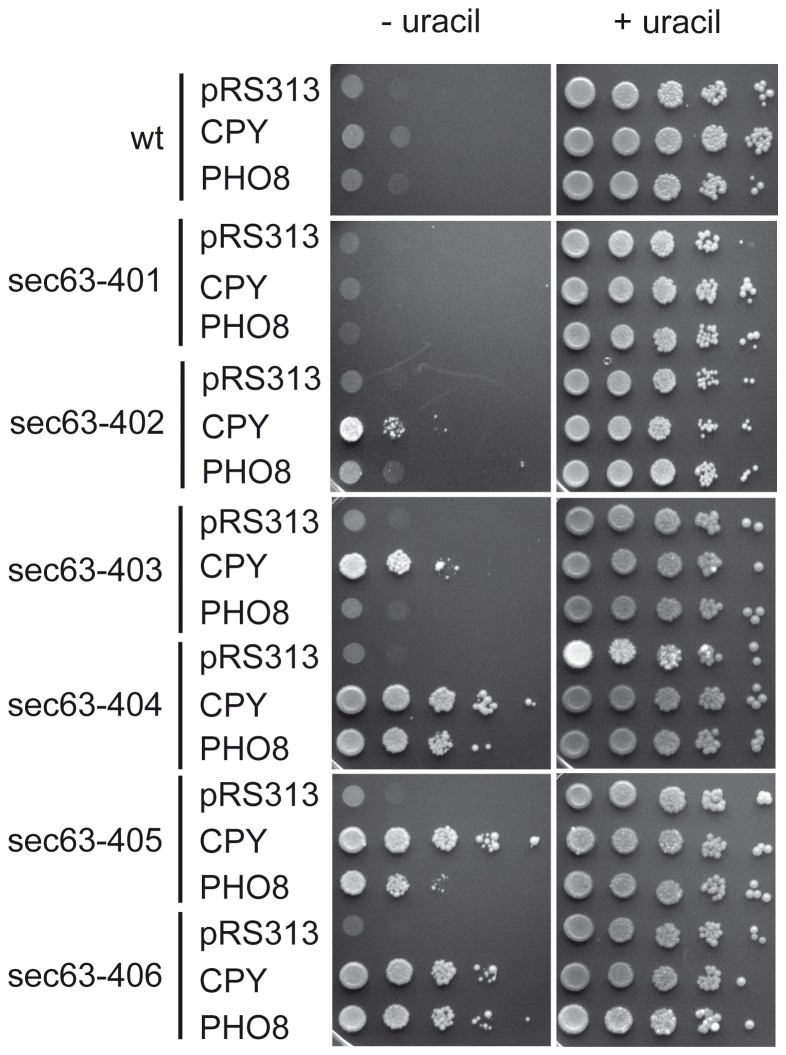
ER protein import defects in the new *sec63* mutants. The *sec63* mutants and W303-1C (wt) were transformed with reporter plasmids for cotranslational import, pRS313-*URA3-PHO8*, and for posttranslational import, pRS313-*URA3-CPY*, or with the empty vector (pRS313). The transformants were grown at 30°C on media lacking histidine and uracil (left) or media lacking histidine (right). Two independent experiments were performed.

Accumulation of misfolded proteins in the ER frequently leads to induction of the UPR, and the UPR is therefore usually activated in ERAD defective yeast strains [35], [36]. We analyzed UPR activity in *sec63-402*, the mutant with the strongest CPY* accumulation, using a *LacZ*-reporter under the control of the UPR element (UPRE) from Kar2p [37]. As a control construct we used a *LacZ* plasmid without the UPRE [37]. Further controls were the *SEC63* wildtype strain expressing the *prc1-1* allele encoding CPY* to exclude a possible influence on the UPR by the expression of misfolded CPY*. As a positive control we used *sec61-32*, the *sec61* mutant with the strongest reported ERAD defect so far [19]. In our hands the UPR was only slightly activated in *sec61-32* (Fig. 3). In *sec63-402* cells, however, the UPR was about 4 times higher than in wildtype. Maximal UPR induction in this strain by treatment with tunicamycin was 4.5 times higher than wildtype (not shown). In *sec63-404*, the mutant with the strongest temperature sensitivity (Fig. 2, right) and strong import defects into the ER (Fig. 4), the UPR was also activated, but not as to the same degree as in *sec63-402* (Fig. 3). This data shows that the D140V mutation in the DnaJ-domain of Sec63p in *sec63-402* has a profound effect on ER protein homeostasis.

### Mutations in Different Sec63p Domains Differentially Affect Co- and Posttranslational Import into the ER

Sec63p is essential for both posttranslational and cotranslational protein import into the ER [3], [16]. We therefore investigated defects in protein import into the ER in our *sec63* mutants using a reporter system [38]. In this system the *URA3* reporter gene is fused to the signal anchor of Pho8p for examination of cotranslational import, and to the signal sequence of CPY for posttranslational import [38]. In wildtype cells Ura3p is imported into the ER which makes it unable to perform its enzymatic role in uracil biosynthesis in the cytosol and the strain is auxotroph for uracil [38]. If the ER import pathway relevant for the fused signal peptide is defective, Ura3p remains in the cytoplasm and the strain becomes prototroph for uracil [38]. As shown in Fig. 4, *sec63-402* and *sec63-403* displayed slight posttranslational import defects. The other DnaJ domain mutant, *sec63-401*, was not impaired in protein import into the ER in this assay (Fig. 4). We observed very strong co- and posttranslational import defects in *sec63-404* and *sec63-406,* and a strong posttranslational, but more modest cotranslational import defect in *sec63-405* (Fig. 4).

To narrow down the observed defects to distinct Sec63p domains we also tested protein import into the ER in the mutants with mutations in individual Sec63p domains (Fig. S2). In *sec63-404* mutations in both the Brl domain and the acidic domain contributed to the observed defects in co- and posttranslational import into the ER (Fig. S2). Our results regarding the Brl domain are in agreement with Jermy and colleagues who had published that the deletion of amino acids 550-611, which are located at the C-terminal region of the Brl domain, and where two mutations are located in sec63-404, causes a loss of both cotranslational and posttranslational import [39]. In *sec63-404*, however, the mutations in the acidic domain had a stronger effect on both co- and posttranslational import into the ER than those in the Brl domain, and in *sec63-406* the mutations in the acidic domain were solely responsible for both types of import defect (Fig. S2). These results were unexpected as the acidic domain had previously been identified as interacting with Sec62p which was only known to be required for posttranslational import, and deletion of the entire acidic domain only marginally affected cotranslational import [29], [39]. Sec62p has, however, recently been shown to also be important for cotranslational insertion and orientation of moderately hydrophobic signal anchors [40]. Although signal-anchored wildtype Pho8p insertion into the ER membrane is independent of Sec62p [40] the Pho8-URA3p reporter protein used in the experiments shown in Fig. 4 may require Sec62p for membrane insertion which would explain its dependence on the acidic domain of Sec63p. In *sec63-405* the mutation in the first transmembrane domain caused the strong posttranslational import defect whereas the mutation in the Brl domain barely affected import (Fig. S2). Our data confirm that the C-terminal part of the Brl domain plays an essential role for co- and posttranslational import, and demonstrate that the acidic domain at the Sec63p C-terminus is also required for both import pathways. We also show that mutations in the Sec63p DnaJ domain, TMD1, and the N-terminal part of the Brl domain primarily influence the posttranslational import.

### CPY* ERAD is Compromised in *sec63-402* Yeast

**Figure 5:**
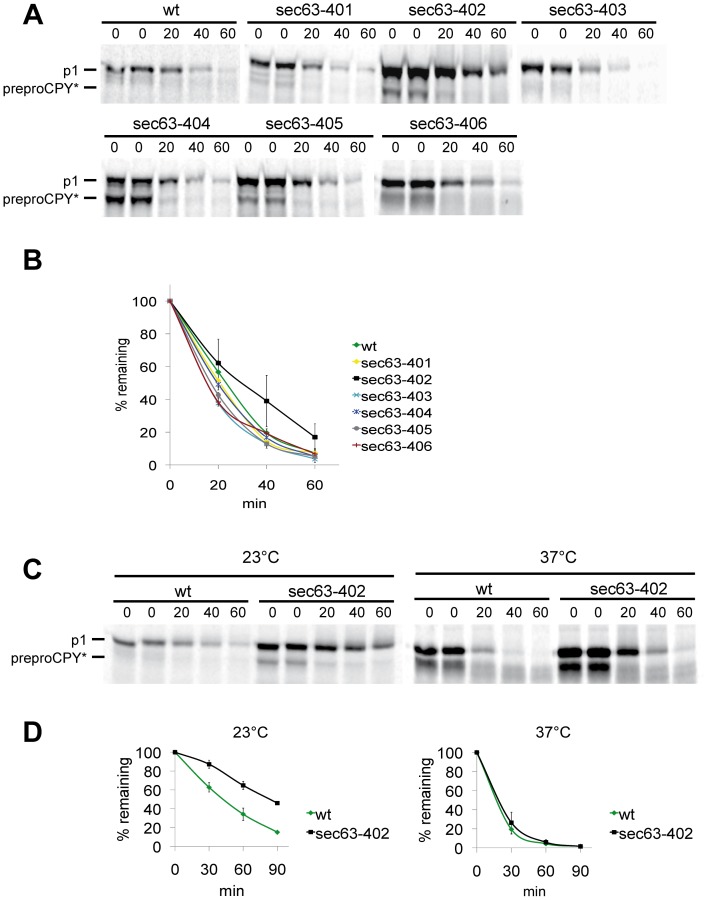
CPY* is stabilized in *sec63-402*. A: CPY* degradation was examined by pulse chase analysis in all new *sec63* mutants and the corresponding wildtype. Cells were grown at 30°C to early log phase and labeled with [^35^S] methionine/cysteine for 2 min, followed by a chase for the indicated times. Cells were lysed and CPY* immunoprecipitated and analysed on 10% gel SDS-gels and detected by autoradiography. B: CPY* was quantified using a phosphorimager; the results of 3 independent experiments are shown in the graph. C: CPY* degradation in *sec63-402* at different temperatures; wildtype and *sec63-402* were grown at 30°C to early log phase, then cells were transferred to 23°C or 37°C. D: Quantitation of CPY* from the experiments shown in C. Mean values of 2 independent experiments are shown.

In order to more closely examine the degradation defects for CPY* in our *sec63* mutants, the mutants were pulse-labeled for 2 min, chased for the indicated times, and levels of CPY* examined by immunoprecipitation with a polyclonal antibody and autoradiography. In *sec63-402*, *sec63-404*, *sec63-405* and in *sec63-406* the posttranslational import defect observed with the reporter constructs (Fig. 4) could be confirmed by detection of cytosolic preproCPY* in the immunoprecipitates (Fig. 5). The relative strengths of the posttranslational defects seen here differed, however, from those seen with the reporter constructs (compare CPY-URA3 for *sec63-403*, *sec63-404* and *sec63-405* in Fig. 4 with preproCPY* for *sec63-403, sec63-404* and *sec63-405* in Fig. 5A). The only *sec63* mutant in which CPY* degradation was slowed down at 30°C was *sec63-402* (Fig. 5A). The t_1/2_ of CPY* in *sec63-402* was increased to about 35 min compared to wildtype (20 min) (Fig. 5B). As *sec63-402* displays slight growth defects at 20°C and 37°C (see Fig. 2) the pulse-chase experiment to monitor CPY* degradation was repeated at 23°C and 37°C (Figs. 5C and 5D). At 23°C the t_1/2_ of CPY* was doubled to 83 min in *sec63-402* in comparison to wildtype (43 min) (Fig. 5C). At 37°C the difference in CPY* ERAD between *sec63-402* and the wildtype was minimal, and the ERAD substrate was degraded very rapidly in both strains with a t_1/2_ of 12 min (Fig. 5D).

Mades and colleagues published recently that in mammalian cells the expression level of Sec63p influences the steady state levels of multi-spanning membrane proteins with 2, 3, or 4 TMDs [41]. The process Sec63p is involved in mammalian cells is independent of proteasome activity and therefore clearly distinct from ERAD, but it is partially dependendent on the Sec63p J-domain [41]. We therefore investigated ERAD in *sec63-402* cells of different substrates that were either soluble (CPY* and KHN) [42], or had 2 (Deg1::Sec62^ProtA^) [43], [44], or 10 transmembrane domains (Sec61-2p) [22], [45]. KHN is a virus haemagglutinin neuraminidase fused to the signal sequence of Kar2p [42]. This substrate had an increased t_1/2_ from 35 min (wildtype) to 45 min in *sec63-402* cells (Fig. S3). Since Mades and colleagues had observed an effect of *SEC63* overexpression on Sec62p levels in mammalian cells we next chose to investigate the fate of the transmembrane substrate Deg1::Sec62^ProtA^, which is a Sec62p construct with an N-terminal degradation sequence [43], [44]. ERAD of this substrate is initiated by translocation of its cytosolic N-terminus into the ER and its N-glycosylation resulting in a molecular weight increase [43]. Degradation then proceeds in dependence on the ER-resident E3 ubiquitin ligase Hrd 1p [46]. If posttranslational import of the Deg1::Sec62^ProtA^ into the ER is delayed, N-glycosylation is also delayed, and the unglycosylated faster migrating band becomes predominant on the gel. At the same time, degradation of the protein is switched to a pathway involving the Doa10p ubiquitin ligase and accelerated degradation [46]. As shown in Fig. S4A Deg1::Sec62^ProtA^ degradation proceeded with identical kinetics in wildtype and *sec63-402* cells, and the N-glycosylated form of Deg1::Sec62^ProtA^ was the dominant band in both strains (Fig. S4A, left and middle panels). In contrast, when we performed the experiment in a mutant with a strong posttranslational import defect, *sec63-404 Brl*, we observed a doublet for Deg1::Sec62^ProtA^ with a dominant lower, unglycosylated band indicative of a delay of ER-import of the N-terminus, and acceleration of degradation indicative of a switch to Doa10p-dependent degradation (Fig. S4A, right panel; [46]). Our data show clearly, that there is no effect of *sec63-402* on ERAD of Deg1::Sec62^ProtA^.

We also examined ERAD of Sec61-2p, which carries a point mutation in transmembrane domain 5 of the protein, in our *sec63* mutants [22]. Unfortunately, however, it appears that the interaction of Sec63p and Sec61p, Sec63p and Sec62p, or the lack thereof in specific mutants influenced the results of this experiment. The Brl domain mutations which reduce Sec61 complex/Sec63p interaction, for example, resulted in reduced Sec61-2p ERAD whereas the acidic domain mutations which ablate interaction with Sec62p accelerate it (Fig. S4B). The fact that *sec63-402* accelerates Sec61-2p degradation is difficult to explain, but it is the opposite effect to that observed by Mades et al. where a J-domain mutation in the overexpressed *SEC63* led to a partial stabilization of multi-spanning membrane proteins [41].

In addition, we examined the effects of SEC63 expression levels on ERAD in yeast. Overexpression (6-fold) of Sec63p resulted in a marginal increase of CPY* degradation compared to wildtype, but had no effect on the turnover of Deg1::Sec62^ProtA^ (not shown). The Sec63p-depleted strain could not be used for evaluation of CPY* degradation as preproCPY* import was nearly completely blocked and cotranslational import into the ER is also compromised under these conditions resulting in reduced viability of that strain (not shown and [4]). We show here that only ERAD of soluble substrates is affected in *sec63-402* cells, suggesting that the role of Sec63p in yeast ER quality control is restricted to ERAD of soluble proteins.

## Discussion

How misfolded proteins are exported from the ER and which proteins are participating in this process is still not understood. Several studies using the *sec63-1* mutant in pulse-chase experiments indicate that Sec63p might be involved in ERAD [19], [20], but because *sec63-1* is leaky it was unclear whether the effects of the mutant on ERAD were due to a direct participation of Sec63p in the process or whether the effects were an indirect consequence of the defective ER structure in the mutant. Sec63p, but none of the other subunits of the Sec63 complex, also prominently copurified with proteasomes attached to the Sec61 channel, but did not contribute to anchoring the proteasome to the ER membrane [23]. This prompted us to reinvestigate the role of Sec63p in ERAD by generating new mutants which were selected in a screen monitoring the intracellular accumulation of the ERAD substrate CPY*. Characterizing these new *sec63* mutants we confirm that Sec63p is a part of the ERAD machinery and demonstrate that only its DnaJ domain is functionally important for ERAD.

Colony blotting has been used successfully before to identify proteins that take part in the degradation of misfolded secretory proteins [24]. We used this method to isolate mutants with one to five point mutations in different domains of Sec63p (Fig. 1A). Cells expressing the DnaJ domain mutant *sec63-402*, however, were the only ones that significantly accumulated the ERAD substrate CPY* (Fig. 1B). The second DnaJ domain mutant, *sec63-401*, showed only moderate accumulation of CPY* compared to wildtype (Fig. 1A, B). Only the *sec63-402* mutant was sensitive to ER stress induced by the glycosylation inhibitor tunicamycin, and its sensitivity was higher than that of the *sec63-1* mutant (Fig. 2). Tunicamycin sensitivity often indicates the accumulation of misfolded proteins in the ER which in turn elicit the UPR. The UPR was also activated strongly in *sec63-402* confirming a defect in ER protein homeostasis (Fig. 3). The growth of this mutant was compromised mildly at 37°C, and strongly at 20°C, but at 30°C it grew like wildtype, and displayed only a mild posttranslational import defect (Figs. 2, 4, 5). Although some of the other *sec63* mutants had much more substantial co- and posttranslational import defects (Figs. 4, 5), *sec63-402* was the only one of our mutants defective in degradation of the soluble ERAD substrate CPY* at 30°C, and the ERAD defect was exacerbated at lower temperature (Fig. 5). The two-fold increase in half-life of CPY* at 30°C was comparable to the ERAD defects observed previously for *sec63-1* in vivo for CPY* and in a cell-free ERAD assay for ▵gpaF [19], [20].

The temperature sensitivity in *sec63-402* was very mild but it was the only cold-sensitive mutant (Fig. 2). At 37°C *sec63-404* and *sec63-406* which have several point mutations in the acidic domain and the C-terminal part of the Brl domain displayed particularly severe growth defects (Fig. 2). These mutants also had the strongest co- and posttranslational protein import defects into the ER (Fig. 4).

The last 14 amino acids of the acidic domain are essential for the interaction with Sec62p, whereas the Brl domain interacts with the Sec61 complex [29], [39]. Both *sec63-404* and *sec63-406* carry one mutation in the Sec62p interaction area (Fig. 1A, asterisks). In addition, *sec63-404* has three mutations in the C-terminal part of the Brl domain, whereas *sec63-406* has one more mutation in the acidic domain and two further mutations in the Brl domain (Fig. 1A). The *sec63-404* mutant was unable to grow at 37°C, whereas *sec63-406* cells were viable but grew slower than wildtype (Fig. 2). The mutations in the acidic domain caused the temperature sensitivity in *sec63-406,* whereas in *sec63-404* both Brl and acidic domain mutations contributed to the growth defect at 37°C (Fig. S1). Our data in agreement with previous publications demonstrate that interactions of Sec63p mutated in the Brl and acidic domains with Sec61p and Sec62p become unstable at higher temperatures, and that this affects both modes of protein import into the ER and hence viability (Figs 1A, 2, 4, S1, S2) [32], [47].

It is well established that Sec63p is involved in co- and posttranslational protein import [3], [4], [16]. Contributions of its individual domains to protein import into the ER are distinct: We show here that the DnaJ domain is only required for posttranslational import as shown by the exclusively posttranslational import defect in *sec63-402* (Fig. 4). In contrast the Brl domain and the acidic domain are involved both in post- and in cotranslational import (Figs 4, S1). In *sec63-406* the two mutations in the acidic domain (one in the Sec62p interaction region, one more N-terminal) are almost exclusively responsible for the co- and posttranslational import defects observed in this mutant (Fig. S2), whereas in *sec63-404* the mutant Brl and acidic domains both independently contribute to the import defects observed in this mutant (Figs. 4 and S2). Mutations in the Brl domain appear to have stronger effects on posttranslational import than on cotranslational import (Fig. S2) which may indicate that interaction of the Sec63p Brl domain with the Sec61 complex is more important in formation of a functional heptameric Sec complex than in presence of the ribosome during cotranslational import. Similarly the mutation in the first Sec63p transmembrane domain in *sec63-405* causes a stronger defect in posttranslational import, perhaps suggesting that this domain contributes to stabilization of the heptameric Sec complex during posttranslational import.

The posttranslational protein import defects observed with the URA3 reporter assays were confirmed in the pulse-chase of CPY*, but the relative strengths of the defects were different in both experiments (compare growth of CPY-URA3 in Fig. 4 with accumulation of preproCPY* in Fig. 5A for *sec63-403, -404, -405*). This might be a kinetic effect as CPY* was labeled for 2 min only in the pulse, and precursor accumulation in this experiment therefore reflects the lack of import of newly synthesized protein only, whereas the CPY-URA3 reporter assay reflects the steady state levels of cytosolic precursor accumulation due to the import defects.

The DnaJ domain named after the *E. coli* Hsp40 DnaJ is highly conserved and acts as a cochaperone for Hsp70 proteins [48], [49]. Interaction of Hsp70s with DnaJ domains leads to ATP hydrolysis by the Hsp70s which results in altered Hsp70 substrate interaction [49]. J-domains consist of four helical domains connected by loops [49]. Essential for the interaction between a J-domain and an Hsp70 protein is the HPD motif located in the loop between the second and the third helix [16], [49], [50]. In *sec63-1* the alanine at position 179 in helix 3 of the Sec63p J-domain is replaced by a threonine [32]. This position is extremely highly conserved and important for helix packing and J-domain stability [49]. The mutations in the Sec63p J-domain in *sec63-401* and *sec63-402* are located in the second helix which is characterized by a lysine-rich surface. The substitution of serine 143 in *sec63-401* with phenylalanine had no striking effect on Sec63p function (Figs. 1A, 2, 4, 5). Position 143 in helix 2 of the J-domain is occupied by a lysine in most J-proteins in yeast including Jem1p, but can also be a serine (Sec63p, Scj1p), threonine, or isoleucine [49]. In *sec63-402* aspartate 140 is replaced by a hydrophobic valine (Fig. 1A). Most other J-domain proteins, including the other two ER-resident J-proteins in yeast, Jem1p and Scj1p, also have an acidic residue at this position [49]. Residue 140 is located adjacent to a highly conserved, functionally important isoleucine that is essential for helix packing and stabilization of the J-domain [49]. The D140V mutation in *sec63-402* may disturb the conformation of the second J-domain helix and thus alter the position of the HPD motif; alternatively, the substitution may interfere with the bending of helix II upon interaction with Kar2p [49], [51].

The functions of the J-domain proteins involved in ERAD are distinct: While Jem1p and Scj1p in cooperation with Kar2p prevent soluble misfolded protein aggregation and thus keep soluble ERAD substrates in an export-competent state, Sec63p is a typical type III J-domain protein which recruits Kar2p to a specific site, the translocon in the ER membrane [22], [49], [52]. Sec63p/Kar2p promote transport of proteins through the Sec61 channel into the ER, and our data suggest that Sec63p/Kar2p are also required for soluble misfolded protein export from the ER to the cytosol [15], [16]. Our data seemingly contradict work by Vembar et al. (2010) who showed that a mutation in the Kar2p J-domain interacting surface (R217A) which reduced the affinity of Kar2p for Sec63p, but not for Jem1p, was without effect on ERAD of two soluble substrates, CPY* and ▵gpaF [53]. A mutation in the substrate interaction domain of Kar2p, on the other hand, was defective in degradation of both substrates [53]. These observations, however, can be reconciled with ours: For import into the ER the Sec63p/Kar2p interaction is limiting because tethering of Kar2p to the protein translocation channel keeps the chaperone in close proximity to the translocating substrate, hence Kar2p affinity for the substrate is less critical. For soluble protein export to the cytosol the Kar2p/export substrate interaction is limiting because reduced substrate binding to Kar2p results in loss of export competence, substrate aggregation, and an ERAD defect. Even in this scenario one would expect a limited effect on ERAD of the reduced affinity of Kar2p for Sec63p in the R217A mutant. In the analysis of ERAD in this mutant Vembar et al. chose a very long pulse (10 min) compared to ours (2 min), and the t_1/2_ of CPY* in their wildtype was unusually long (40 min) compared to the literature and our experiments (20 min), so modest effects on CPY* ERAD might not have been detected in this experiment [53]. The authors also monitored ▵gpaF degradation in a cell-free system and found no defect in *kar2R217A* membranes [53]. In this in vitro assay the import reaction to load the microsomes with ▵gpaF is much longer (1h) than the time required to complete import (10-15 min), and this prolonged import reaction is essential for subsequent export and degradation in the presence of cytosol [18], [19], [54]. If the purpose of the Sec63p/Kar2p interaction in ERAD is targeting substrates to the export machinery, targeting may have already been completed during the import reaction in the cell-free ERAD assay. Alternatively, in the absence of competing import into the ER, Sec63p interaction may not be limiting for ERAD in vitro, and hence the modest reduction in the Sec63p/Kar2p interaction in *kar2R217A* membranes may not manifest itself as export defect [53]. On the whole these experiments illustrate the problems in comparing roles of non-essential and essential J-domain proteins in ERAD by comparing deletions of the former with point mutations in the latter, which by definition can never be completely dysfunctional and hence are bound to present with more modest phenotypes.

The *sec63-402* mutant was the only one of our new *sec63* mutants that displayed a significant ERAD defect, and we only observed a defect in degradation of soluble ERAD substrates (CPY*, KHN), not with transmembrane ERAD substrates (Deg1::Sec62^ProtA^, Sec61-2p) (Figs. 5, S3, S4). This was in apparent contrast to the results from Mades et al. [41] who had shown that in mammalian cells the steady state levels of transmembrane proteins were inversely correlated with the Sec63p expression level. The process described by Mades et al., however, was independent of proteasome activity, and hence not mediated by ERAD [41]. In mammalian cells, Sec63p influences multispanning membrane protein expression levels during biosynthesis only [41], suggesting that it may have a quality control function during membrane integration of multi-spanning proteins. Since in mammalian cells the coupling between translation and translocation is much tighter than in yeast, aborted membrane integration in *SEC63*-overexpressing cells would result in the protein never being fully translated and reduced expression levels independently of proteasome activity. We also examined the effects of Sec63p expression levels on ERAD. Overexpression (6-fold) of Sec63p resulted in a marginal increase of soluble CPY* degradation compared to wildtype and had no effect on polytopic transmembrane Deg1::Sec62^ProtA^ degradation (not shown). In contrast to mammalian cells in which Sec63p can be completely depleted [41], in yeast Sec63p depletion affected viability of the cells and could therefore not be evaluated for effects on ERAD (not shown). Our data and that of Mades et al. [41] indicate that the functions of Sec63p in ER protein quality control differ between yeast and mammalian cells: In mammals, Sec63p affects the biosynthesis of multi-spanning membrane proteins independently of proteasome activity and hence ERAD, whereas in yeast Sec63p is required for ERAD of soluble proteins. Thus the function of Sec63p in the ER must have changed during evolution. Our work therefore suggests that yeast is not an appropriate model organism to study diseases that are caused by mutations in or altered expression of mammalian *SEC63* (polycystic liver disease, certain cancers) [8], [55].

## Materials and Methods

### Yeast Strains, Plasmids and Antibody

The W303-1C (*MATα ade2-1 ura3-1 his3-11,15 leu2-3,112 trp1-1 can1-100 prc1-1*) and the strain W303-1A (*MATa ade2-1 ura3-1 his3-11,115 leu2-3,112 trp1-1 can1-100*) were used for controls and constructing the *sec63* mutants. As a positive control for the UPR- assay *sec61-32* (*MATα can1-100 leu2-3,112 his3-11,15 trp1-1 ura3-1 ade2-1 sec61::HIS3* [pDQ *sec61-32*]) was used. The *Δder1* strain KRY880 (*ade2-1 ura3-1 his3-11,15 leu2-3,112 trp1-1 can1-100 prc1-1 der1::natNT2*) was constructed by integration of a PCR product of pYMnatNT2 according to Janke et al. [56]. The genotypes of the *sec63* mutants are *MATα ade2-1 ura3-1 his3-11,15 leu2-3,112 trp1-1 can1-100 prc1-1 SEC63::natNT2 sec63-401* to -*406*. The mutants with individual mutated domains were generated as follows: The Brl and the acidic domains from *sec63-404* and *sec63-406* were separated at 1841 bp of *SEC63* by *PstI* which cuts as well in the vector. The 2 parts of the gene were combined with the corresponding parts of *PstI*-cut *wtSEC63-*pRS315 plasmid, respectively. Correct orientation was verified. The transmembrane domains were separated in *sec63-405* at 400 bp by *SpeI* digestion. The vector of the *sec63-405* plasmid was cut with *HindIII,* and the parts of the plasmid were completed with *SpeI/HindIII*-digested *wtSEC63*-pRS315. The plasmids *PHO8-URA3*-pRS313, *CPY-URA3*-pRS313, pJC30 (*LacZ*-pRS314), pJC31 (*UPRE-LacZ*-pRS414), pSM70 (KHN-HA-plasmid) and *sec61-2-HA-URA3*-pRS316 were generously provided by Davis Ng. pPN1992 (*SEC63-URA3*-pRS316), Deg1::Sec62^ProtA^-pRS316 and a polyclonal antibody against Sec62p were provided by Randy Schekman. Antibody against CPY was raised in rabbit against a purified CPY-His6 peptide from an expression construct provided by Colin Stirling. Rabbit anti HA-antibody was obtained from Covance (catalog number: PRB-101C).

### Screen for *sec63* Mutants

W303-1C and W303-1A were crossed and transformed with plasmid *SEC63-URA3*-pRS316. Genomic *SEC63* ORF was replaced by integration of the *natNT2* gene, which is selected by ClonNat [56]. The diploid strain was sporulated (sporulation media: 0,5% glucose, 1% KCl, 0,1% yeast extract) and haploid progeny was isolated that was *prc1-1* (CPY* allele; tested by pulse chase; see below), *SEC63*-*URA3*-pRS316 and *natNT2* by d/o Ura media including ClonNat. This haploid strain was transformed with mutagenized *sec63-LEU2*-pRS315 plasmids, that had been generated by PCR of *SEC63* with 2 mM of dGTP and dATP and 10 mM of dCTP and dTTP as well as 7 mM MgCl_2_ to insert random point mutations into the *SEC63* gene (primers used: ep_rev: 5` c cga cgg agc tcg ctc atg gct tcg aac aag tgg 3` and ep_for: 5` cg gcc gga tcc gga aac ctt gca atc agt agt gg 3`). The PCR-products were pooled and cloned in the pRS315 vector using *BamH*I and *Sac*I restriction sites. The transformants were grown on 5-FOA plates to allow plasmid shuffling. The new *sec63* mutants were selected by colony blotting [24]. For this the colonies were grown on nitrocellulose membrane for 1 day at 30°C. The membrane was placed onto sporulation media and incubated overnight. After inhibiting protein biosynthesis by incubation on sporulation media including 4 µg/ml cycloheximide for 10 h at 30°C, the cells were lysed and CPY* was detected by immunostaining. Clones forming dark colonies were selected for further analysis.

### UPR-Activity Assay

Reporter plasmid pJC31 (*UPRE-lacZ*-pRS314) and control plasmid pJC30 (*lacZ*-pRS314) were transformed into yeast. The cells were grown to 0.5 OD_600_ and 1 OD_600_ was removed, centrifuged and resuspended in 1 ml Z-buffer (60 mM Na_2_HPO_4_, 40 mM NaH_2_PO_4_, 10 mM KCl, 1 mM MgSO_4_, 0,27% β- mercaptoethanol). Chloroform and 0.1% (w/v) SDS were added and the sample was vigorously shaken. The sample was incubated at 28°C and after 5 min 200 µl of the substrate ONPG (4 mg/ml ortho -nitrophenyl- β-D-galactoside; Sigma Aldrich) was added and the reaction was stopped after 20 min by 500 µl 1 M Na_2_CO_3_. Cell debris was removed by centrifugation and the supernatant was examined photometrically at 420 nm. Miller units were calculated: 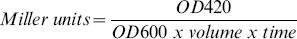



(OD_420_: measured value of ONPG; OD_600_: measured OD of the culture at assay start; volume: used volume of the culture for assay; time: min)

### Cell Labeling and Immunoprecipitation

1.5 OD_600_ per aliquot of early log cells were incubated in synthetic media lacking methionine, cysteine and (NH_4_)_2_SO_4_ for 20 min at the appropriate temperature. The cells were labeled with 50 µCi [^35^S] methionine/cysteine (PerkinElmer) mix for 2 min. Chase was started by adding 0.03% cysteine, 0.04% methionine and 10 mM (NH_4_)_2_SO_4_ to each aliquot. The chase was stopped by adding cold 20 mM Tris-HCl pH 7.5 with 20 mM NaN_3_. Cells were harvested and incubated in 100 mM Tris-HCl, pH 9.4, for 10 min at room temperature. After centrifugation, the cells were lysed with glassbeads in lysis buffer (20 mM Tris-HCl, pH 7. 5, 2% (w/v) SDS, 1 mM DTT, 1 mM PMSF) and lysate denatured for 5 min at 95°C. The glassbeads were washed and the collected supernatant was used for immunoprecipitation with 60 µl Protein A-Sepharose beads (GE Healthcare) and 10 µl polyclonal rabbit antiserum against CPY. The precipitation was performed for 2 h at room temperature or overnight at 4°C. The Protein A-Sepharose beads were washed as in Baker et al. [57]. The precipitate was eluted by heating for 5 min in sample buffer and analysed by electrophoresis on 10% polyacrylamide SDS gels. The signal was detected by autoradiography on a phosphorimager (Typhoon, GE Healthcare) and quantitation was performed with ImageQuant TL (GE Healthcare).

### Cycloheximide Chase

Cells from an overnight culture were grown at 30°C to 1 OD_600_ in SD media with 2% galactose and 2% sucrose. Cycloheximide was added to a final concentration of 200 µg/ml to inhibit the protein synthesis. At the indicated time points 2 OD_600_ was removed and stored in liquid nitrogen. Thawed cells were washed with sterile water, lysed with glassbeads, heated for 5 min at 95°C and centrifuged at 13.000 rpm for 10 min. The supernatant was analysed by electrophoresis on a 12.5% acrylamide gel and Western Blotting. The protein was detected with an antibody against Sec62p.

## Supporting Information

Figure S1 **Temperature- & tunicamycin sensitivity of the** ***sec63*** **mutants with mutations in individual domains.** 10^1^-10^4^ cells of *sec63-404*, *sec63-405* and *sec63-406* as well as the mutants with separated mutated domains and the corresponding wildtype (wt) were grown on YPD plates without or with 0.25 µg/ml tunicamycin at the indicated temperatures. Two independent experiments were performed.
(TIF)

Figure S2 **ER protein import defects in the** ***sec63*** **mutants with separated mutated domains.** The mutants with separated mutated domains from *sec63-404, sec63-405* and *sec63-406* were transformed with reporter plasmids for cotranslational import, pRS313-*URA3-PHO8*, and for posttranslational import, pRS313-*URA3-CPY*, or with the empty vector (pRS313). The transformants were grown at 30°C on media lacking histidine and uracil (left) or media lacking histidine (right). Two independent experiments were performed.
(TIF)

Figure S3 **KHN is stabilized in** ***sec63-402*** **.** KHN degradation was examined by pulse chase analysis in *sec63-402* and the corresponding wildtype. Cells were grown at 30°C to early log phase and labeled with [^35^S] methionine/cysteine for 5 min, followed by a chase for the indicated times. Cells were lysed and KHN immunoprecipitated and analysed on 10% gel SDS-gels and detected by autoradiography. KHN was quantified using a phosphorimager; the results of 2 independent experiments are shown in the graph.
(TIF)

Figure S4 **Deg1::Sec62^ProtA^ and Sec61-2p are not stabilized in** ***sec63-402*** **.** A: The degradation of Deg1::Sec62^ProtA^ was analysed by cycloheximide chase in the mutants *sec63-402*, *sec63-404* Brl, and the corresponding wildtype. The cells were grown at 30°C to OD_600_ = 1 in SD media with 2% galactose and 2% sucrose. Cycloheximide was added to a final concentration of 200 µg/ml. At the indicated time points 2 OD_600_ was removed. Cells were lysed, and cell extracts analysed by electrophoresis on a 12.5% gel and western blot. The protein was detected with an antibody against Sec62p, and wildtype Sec62p is shown as a control. The results of 4 independent experiments are shown in the graph. B: Degradation of Sec61-2p was examined by pulse chase in *sec63-402*, *sec63-405* transmembrane domains, *sec63-404* Brl, *sec63-404* acidic domain, and the corresponding wildtype. Cells were grown at 30°C to early log phase and labeled with [^35^S] methionine/cysteine for 5 min, followed by a chase for the indicated times. Cells were lysed and the HA-tagged Sec61-2p was immunoprecipitated, analysed on 10% gel SDS-gels, and detected by autoradiography. B: Sec61-2p was quantified using a phosphorimager; the results of 3 independent experiments are shown in the graph.
(TIF)
