# Supplementary figures and images for: The Sec63p J-Domain Is Required for ERAD of Soluble Proteins in Yeast
Source: PLoS One. 2013 Dec 4;8(12):e82058. doi: 10.1371/journal.pone.0082058 (PMC3852996; doi:10.1371/journal.pone.0082058)

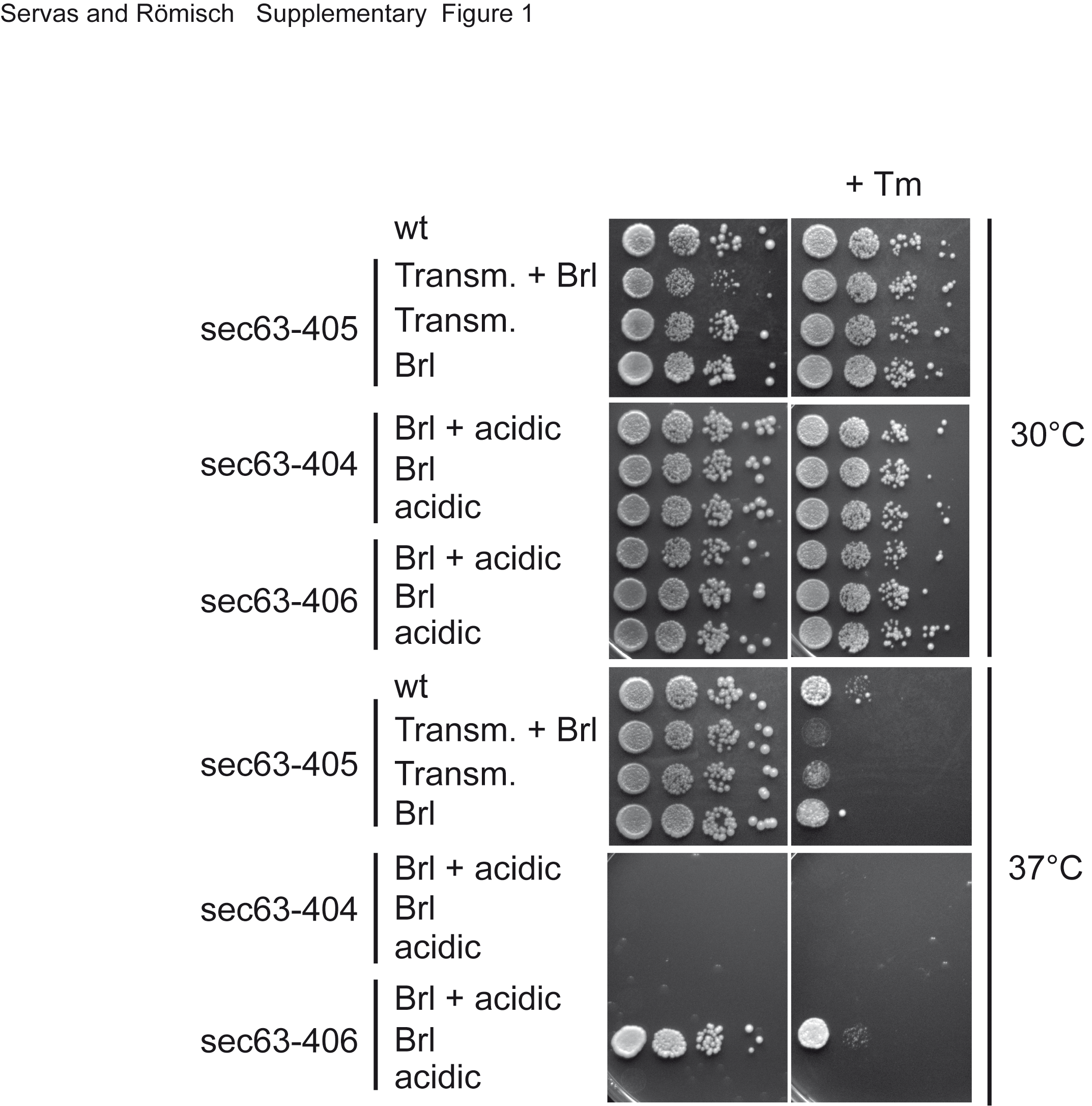

Supplement: Figure S1 — Temperature- & tunicamycin sensitivity of the sec63 mutants with mutations in individual domains. 101-104 cells of sec63-404, sec63-405 and sec63-406 as well as the mutants with separated mutated domains and the corresponding wildtype (wt) were grown on YPD plates without or with 0.25 µg/ml tunicamycin at the indicated temperatures. Two independent experiments were performed. (TIF) [file pone.0082058.s001.tif]

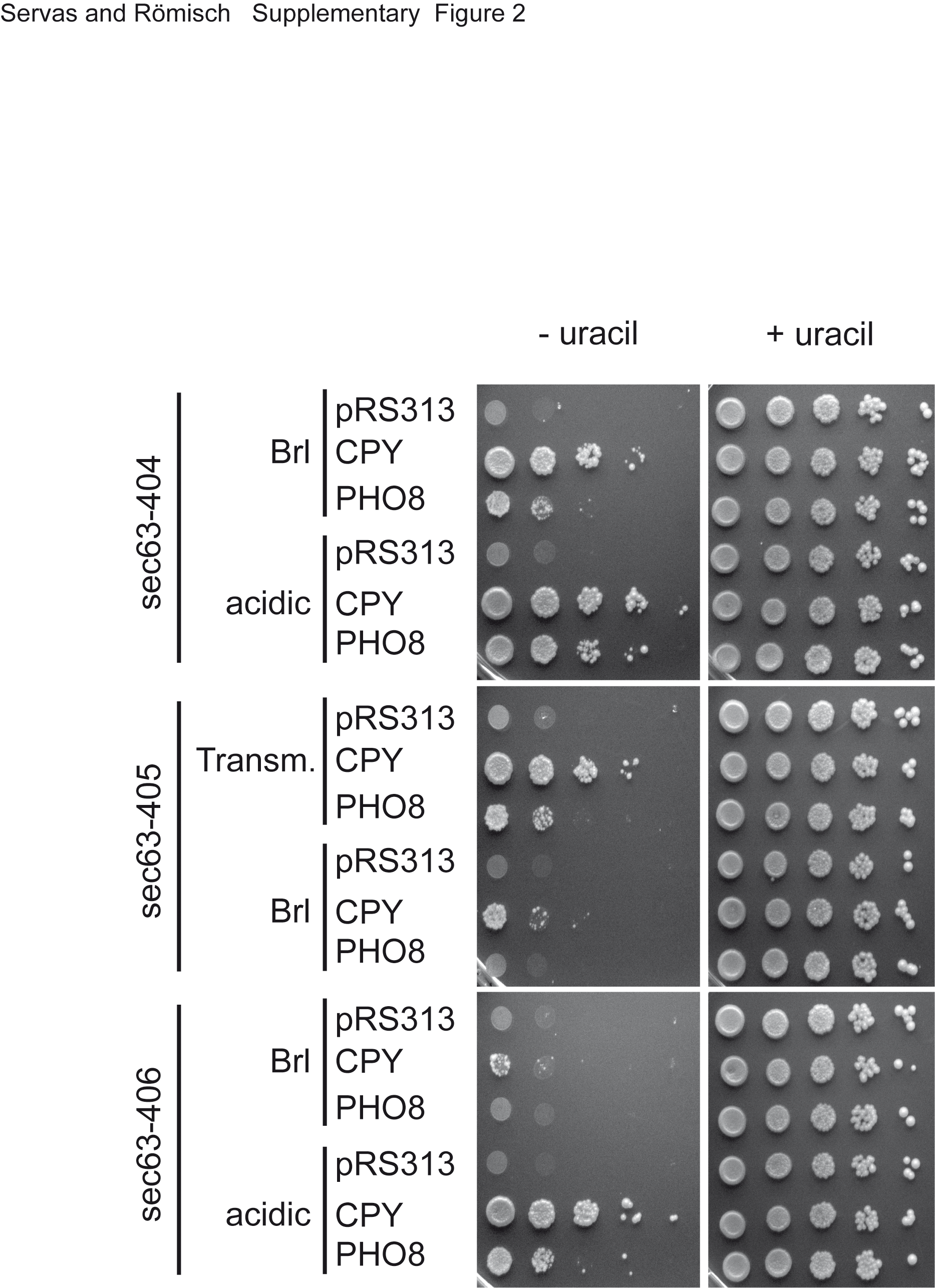

Supplement: Figure S2 — ER protein import defects in the sec63 mutants with separated mutated domains. The mutants with separated mutated domains from sec63-404, sec63-405 and sec63-406 were transformed with reporter plasmids for cotranslational import, pRS313-URA3-PHO8, and for posttranslational import, pRS313-URA3-CPY, or with the empty vector (pRS313). The transformants were grown at 30°C on media lacking histidine and uracil (left) or media lacking histidine (right). Two independent experiments were performed. (TIF) [file pone.0082058.s002.tif]

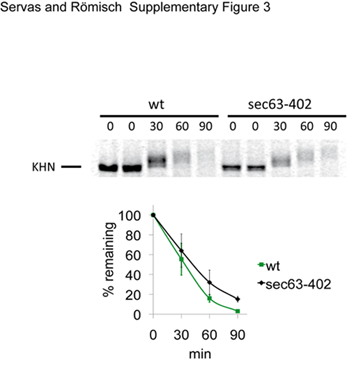

Supplement: Figure S3 — KHN is stabilized in sec63-402 . KHN degradation was examined by pulse chase analysis in sec63-402 and the corresponding wildtype. Cells were grown at 30°C to early log phase and labeled with [35S] methionine/cysteine for 5 min, followed by a chase for the indicated times. Cells were lysed and KHN immunoprecipitated and analysed on 10% gel SDS-gels and detected by autoradiography. KHN was quantified using a phosphorimager; the results of 2 independent experiments are shown in the graph. (TIF) [file pone.0082058.s003.tif]

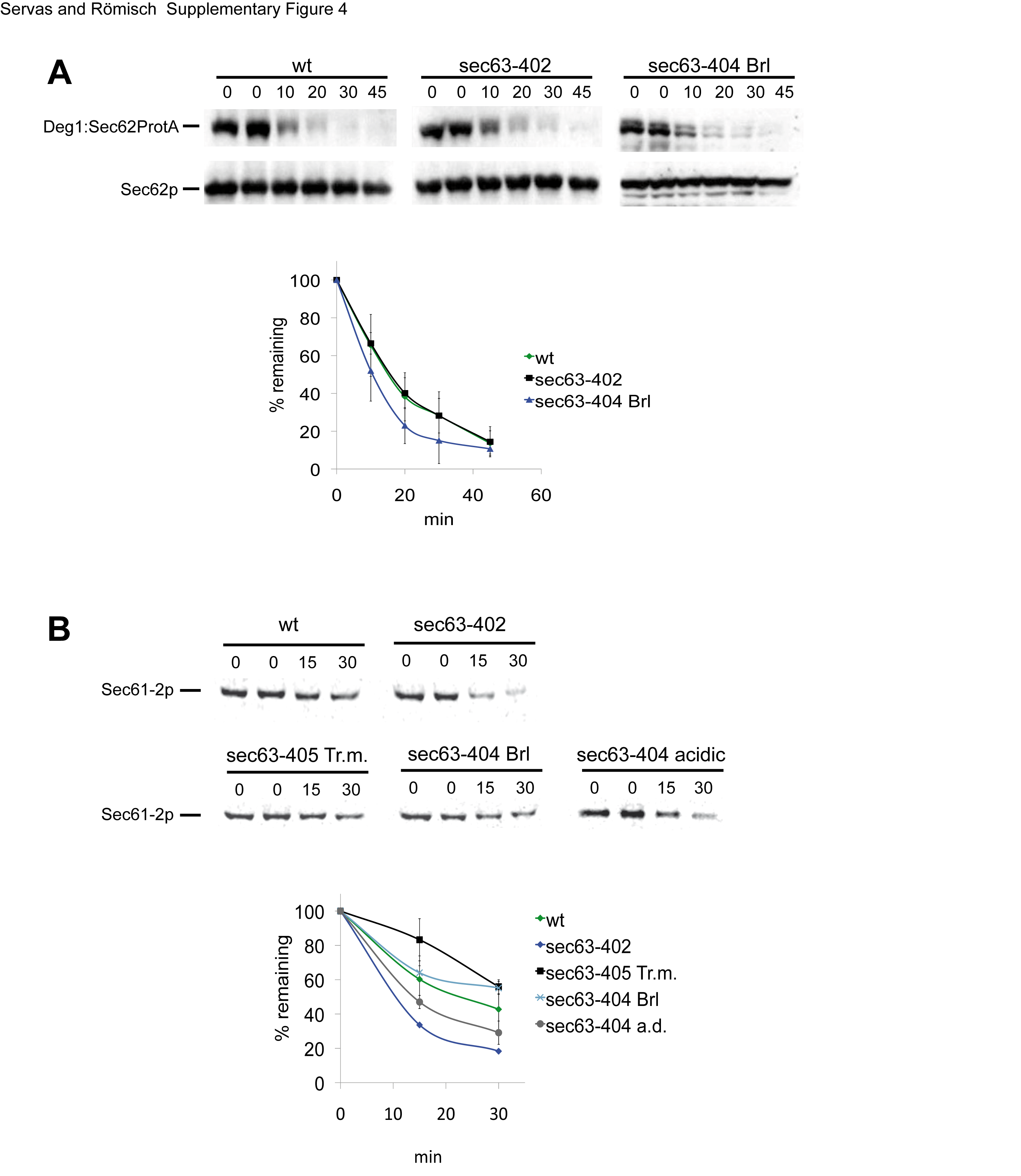

Supplement: Figure S4 — Deg1::Sec62ProtA and Sec61-2p are not stabilized in sec63-402 . A: The degradation of Deg1::Sec62ProtA was analysed by cycloheximide chase in the mutants sec63-402, sec63-404 Brl, and the corresponding wildtype. The cells were grown at 30°C to OD600 = 1 in SD media with 2% galactose and 2% sucrose. Cycloheximide was added to a final concentration of 200 µg/ml. At the indicated time points 2 OD600 was removed. Cells were lysed, and cell extracts analysed by electrophoresis on a 12.5% gel and western blot. The protein was detected with an antibody against Sec62p, and wildtype Sec62p is shown as a control. The results of 4 independent experiments are shown in the graph. B: Degradation of Sec61-2p was examined by pulse chase in sec63-402, sec63-405 transmembrane domains, sec63-404 Brl, sec63-404 acidic domain, and the corresponding wildtype. Cells were grown at 30°C to early log phase and labeled with [35S] methionine/cysteine for 5 min, followed by a chase for the indicated times. Cells were lysed and the HA-tagged Sec61-2p was immunoprecipitated, analysed on 10% gel SDS-gels, and detected by autoradiography. B: Sec61-2p was quantified using a phosphorimager; the results of 3 independent experiments are shown in the graph. (TIF) [file pone.0082058.s004.tif]
